# A single‐cell atlas of bisphenol A (BPA)‐induced testicular injury in mice

**DOI:** 10.1002/ctm2.789

**Published:** 2022-03-28

**Authors:** Siyu Xia, Wei Zhang, Jing Yang, Shuang Wang, Chuanbin Yang, Jigang Wang

**Affiliations:** ^1^ Department of Geriatrics Shenzhen People's Hospital (The Second Clinical Medical College Jinan University The First Affiliated Hospital Southern University of Science and Technology) Shenzhen China; ^2^ Integrated Chinese and Western Medicine Postdoctoral Research Station Jinan University Guangzhou China; ^3^ Artemisinin Research Center and Institute of Chinese Materia Medica China Academy of Chinese Medical Sciences Beijing China; ^4^ Central People's Hospital of Zhanjiang Zhanjiang China; ^5^ Center for Reproductive Medicine Dongguan Maternal and Child Health Care Hospital Southern Medical University Dongguan China; ^6^ National Pharmaceutical Engineering Center for Solid Preparation in Chinese Herbal Medicine Jiangxi University of Chinese Medicine Nanchang China

Dear Editor,

To our knowledge for the first time, we characterized bisphenol A ([2,2‐bis(4‐hydroxyphenyl)propane], BPA), a highly produced chemicals for producing plastics and epoxy resins, induced testicular injury in mice via single‐cell RNA sequencing (ScRNA‐seq). Our high‐resolution cellular atlas not only provides novel insight into the underlying mechanisms and pathways of BPA‐associated testicular injury but also present a valuable resource and foundation for additional discoveries and modifying environmental endocrine disruptors such as BPA‐induced male reproduction toxicity.

BPA is widely employed in multiple consumer products including bottle tops, eyewear, water supply pipes, certain dental sealants and can be found in multiple body fluids including blood, urine of virtually all humans.^1^, ^2^ Due to the potential health effect, the wide environmental exposure BPA has been a great concern. Among all the adverse effects, its potentially harmful effect in the male reproductive system has drawn increasing attention with not fully understood mechanisms though several mechanisms such as impact male germ cells through disruption of Ca^2+^ homeostasis, and promoting Deoxyribonucleic acid (DNA) damage.^1^, ^2^ The increased male reproductive associated disease such as testicular dysgenesis syndrome is associated with increased incidence for exposure to environmental endocrine disruptors such as BPA.^3^ Pre‐puberty is a key stage for male reproductive system development. Adverse pre‐puberty stage injuries/damages caused by exposure to environmental endocrine disruptors including BPA may persist for a long time and even for later adult life.^4^ Notably, accumulating evidence has shown that children are exposed to increasing amounts of BPA,^5^ increasing the great potential reproductive health risk for male children. Thus, an in‐depth examination of the effect of prepubertal BPA exposure by using advanced technologies such as ScRNA‐seq^6^ is important and necessary. Here, we aimed to understand this question in mice.

Here, as shown in the schematic model (Figure 1A) and Figure 1B,C, we found BPA‐induced testicular toxicity as reflected by significantly decreased seminiferous tube diameter, wrinkling on the boundaries of seminiferous tubules, and intensified atrophy. Consistent with previous well‐established findings that BPA affects male reproductive health.^2^ To deeply understand how BPA induces pubertal testicular injury, ScRNA‐seq was performed. Ten cell clusters that represent the major cell types in testis were identified with a pool of 77 776 cells after quality control (Figure S1A,B) and visualized using Uniform Manifold Approximation and Projection (UMAP) algorithm according to canonical cell‐type‐specific marker genes as reported previously (Figure 1D–F, Figure S1C).^7^, ^8^ These cell types are germ cells spermatogonia, meiotic spermatocytes, pachytene, acrosomal, round spermatids (round STids) and elongating spermatids (elongating STids) and somatic cells innate lymph, Telocytes, Leydig, and Sertoli cells. Cell type‐specific gene signatures were shown in Figure S1D and Table S1. Though there was a slight sample to sample variation, BPA specifically increased relatively cell proportions of innate lymphoid cells (Figure 1G and Figure S1E). Overall, we generated a testis representation of cellular diversity and established a cellular roadmap of Sc‐transcriptome map upon BPA treatment in mouse testes.

**FIGURE 1:**
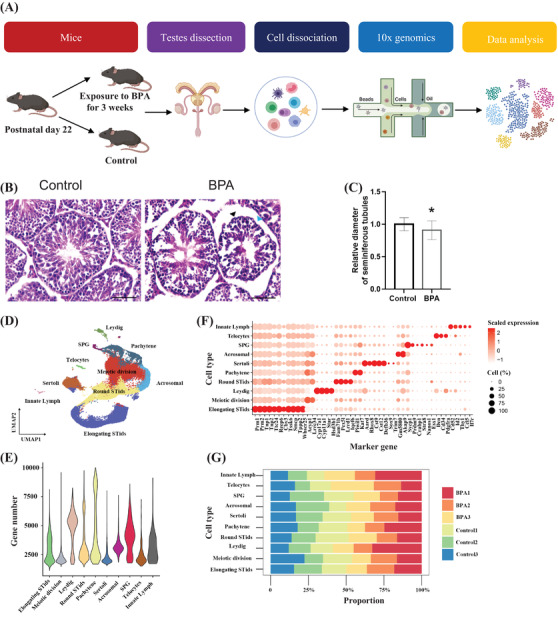
Overall of the experimental design and single‐cell transcriptome analysis of mouse testes upon bisphenol A (BPA) treatment. (A) Schematic model showing the experimental design of this study. (B) Representative Hematoxylin and eosin (H&E) staining images showing BPA‐induced testicular injury in mice. Data were present as mean ± standard error of the mean (SEM) from five mice per group. Wrinkling on the boundaries of seminiferous tubules was shown in black arrows; intensified atrophy and loss of seminiferous tubules were shown in blue arrows. Scale bar 50 μM. (C) Quantification of seminiferous diameter of B. (D) UMAP plot of 77 776 cells from testicular tissue showing 10 major‐coloured clusters of germ cells and somatic cells upon BPA treatment. (E) Violin plot of average gene numbers identified in each cell type. (F) Plot of canonical cell‐type markers in 10 major cell types of the testis. (G) Bar plot of the relative proportions of specified cell types in the testis from untreated and BPA‐treated samples for each cluster

We further discovered highly heterogeneous cell‐type‐specific differential expressed genes (DEGs) in different cell types of BPA‐treated mice (Figure 2A–C, Figure S2A, Table S2). Notably, multiple genes involved in regulating spermatogenesis (Figure S2B, Table S2) such as *Clip1, Cep290*, *Cep126* and *Cep128* were significantly down‐regulated upon BPA treatment (Figure 2C). Several genes such as *Dido1*, *Malat1* and *Rock1* showed bidirectional changes in different cell types (Figure 2C), suggesting that certain changes may be masked by using the traditional bulk RNA‐seq method, and thus our ScRNA‐seq results help to understand the changes in genes expression in an unprecedent resolution upon BPA‐treatment. We then established a comprehensive intercellular network of potential ligand‐receptor interactions and found that the overall interaction numbers and strength within identified cell types upon BPA treatment were increased (Figure 2D‐E). BPA‐increased communication probability of multiple receptor‐ligand pairs in different cell types (Figure 2F). For instance, BPA increased TGFβ1‐(TGFβ1‐TGFβ2) interaction in innate lymph cells, indicating the activation of TGFβ signaling pathways and increased inflammation upon BPA treatment. BPA‐decreased communication probability of multiple receptor‐ligand pairs (Figure 2G). For instance, PDGFA‐PDGFRA interaction, critical for spermatogenesis, in Leydig and pachytene cells was decreased, suggesting a possible role of PDGFA^9^ in BPA‐induced testis injury. Altogether, these findings signify those changes in receptor/ligand interactions may contribute to BPA‐induced perpetual injury.

**FIGURE 2:**
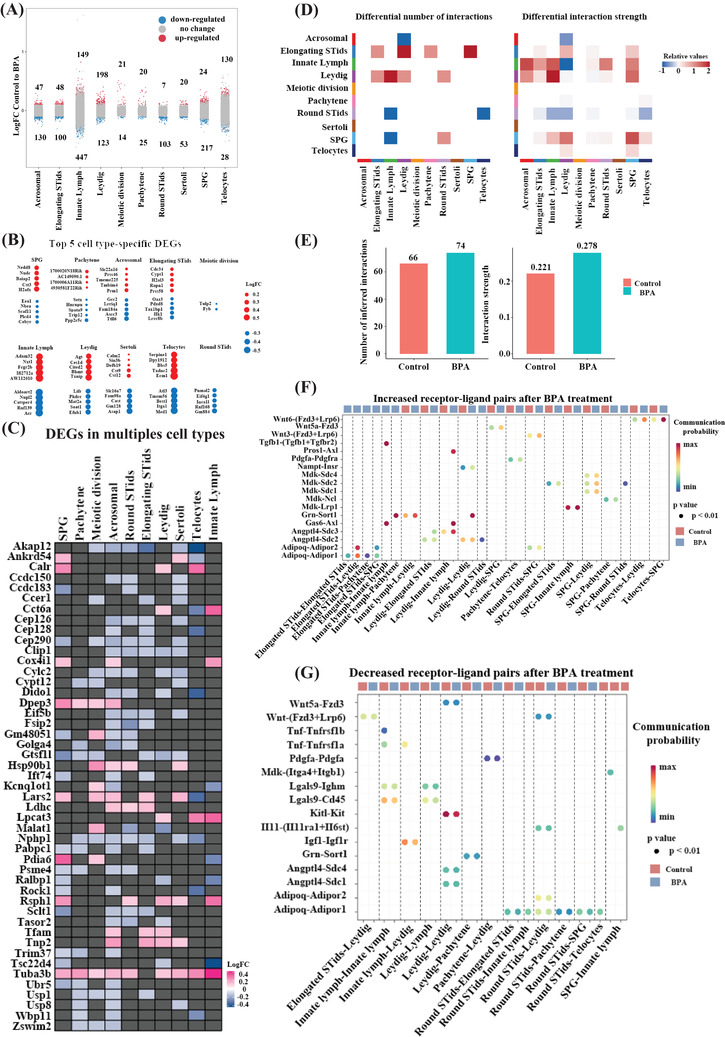
Bisphenol A (BPA)‐associated differently expressed genes (DEGs) in different cell types, and receptor‐ligand pairs changes in the testis. (A) Strip chart showing the BPA‐related changes of all detected genes (dots) across 10 cell types. Red colour showed significantly up‐regulated genes, and blue colour indicated significantly down‐regulated ones. Black colours are not significantly changed upon treatment. (false discovery rate (FDR)  <  .05 and fold change (FC)  >  10%). (B) Cell‐type‐specific top five significantly up‐regulated and down‐regulated genes in different cell types upon BPA treatment. (C) Heat‐map showing differentially expressed genes among different cell types upon BPA‐treatment. (D) Heat‐map plot shows interaction numbers and strength between different cell types upon BPA treatment. (E) Quantification data of interaction numbers and strength in D. **(F)** Bubble plot shows significantly up‐regulated ligand‐receptor pairs upon BPA treatment. **(G)** Bubble plot shows significantly down‐regulated ligand‐receptor pairs upon BPA treatment

We then characterized shared and cell‐type‐specific DEGs associated pathways in somatic cells (Figure 3A–C) and germ cells (Figure 3E–G) upon BPA treatment (Table S3). Gene Ontology (GO) analysis showed top up‐regulated pathways and down‐regulated pathways in different somatic cells and germ cells. Experimental results in increased apoptotic marker Caspase 3 (Figure 3D) and DNA damage marker γ‐H2AX (Figure 3G) further supporting ScRNA‐seq results. These results suggest that multiple shared and cell‐type‐specific pathways such as increased ‘protein folding’ in certain somatic cells, and down‐regulated ‘RNA splicing’ and ‘mRNA catabolic process’ in specific germ cells may be involved in BPA‐induced testicular injury.

**FIGURE 3:**
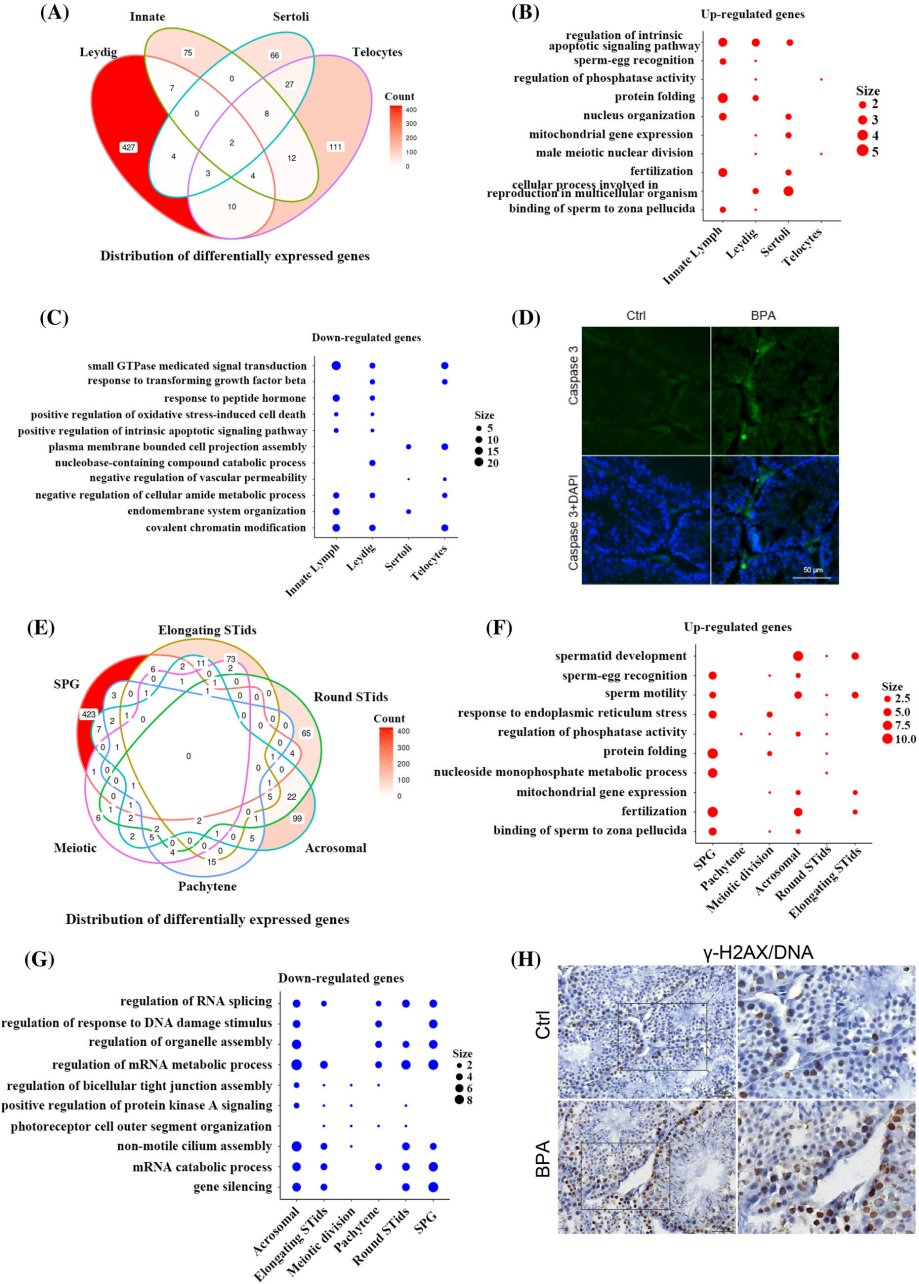
The changes in cellular pathways and processes upon bisphenol A (BPA)‐treatment. (A) Venn diagram showing the distribution of overlapped differentially expressed genes (DEGs) among somatic cell types in the testis upon BPA treatment. (B–C) Representative shared GO terms of up‐regulated (B) and down‐regulated (C) differentially expressed genes in different somatic cell types in the testis upon BPA treatment. (D) Immunofluorescence results showing increased proapoptotic marker Caspase 3. (E) Venn diagram showing the distribution of overlapped DEGs across all the germ cell types in the testis upon BPA treatment. (F–G) Representative shared GO terms of up‐regulated (F) and down‐regulated (G) differentially expressed genes in different germ cell types in the testis upon BPA treatment. (H) Immunofluorescence results showing increased DNA damage marker γ‐H2AX. Size indicates gene number. Data are present as mean ± SEM of three control and three BPA treated testis; *p‐value  <  .05 and FC > 10% indicate significantly differentially expressed genes and significant GO terms

Because Leydig cells are critical in spermatogenesis, we further characterized BPA‐associated molecular alterations in Leydig cells. GO analysis showed multiple up‐regulated gene associated pathways (e.g., ‘response to ER stress response’, ‘cell redox homeostasis), and down‐regulated genes associated pathways (e.g., ‘sterol homeostasis’, ‘cholesterol metabolic process’) (Figure 4A,B) and gene set scores for several key processes including ‘sterol metabolic process’ was significantly decreased (Figure 4C). These results highlighted critical roles of these pathways in Leydig cells for BPA‐induced testicular injury.^10^ We then showed that BPA‐associated‐changes in transcriptional factors (TFs) and their regulated DEGs (Table S4), indicating critical roles of these TFs in BPA‐associated testis injury.

**FIGURE 4:**
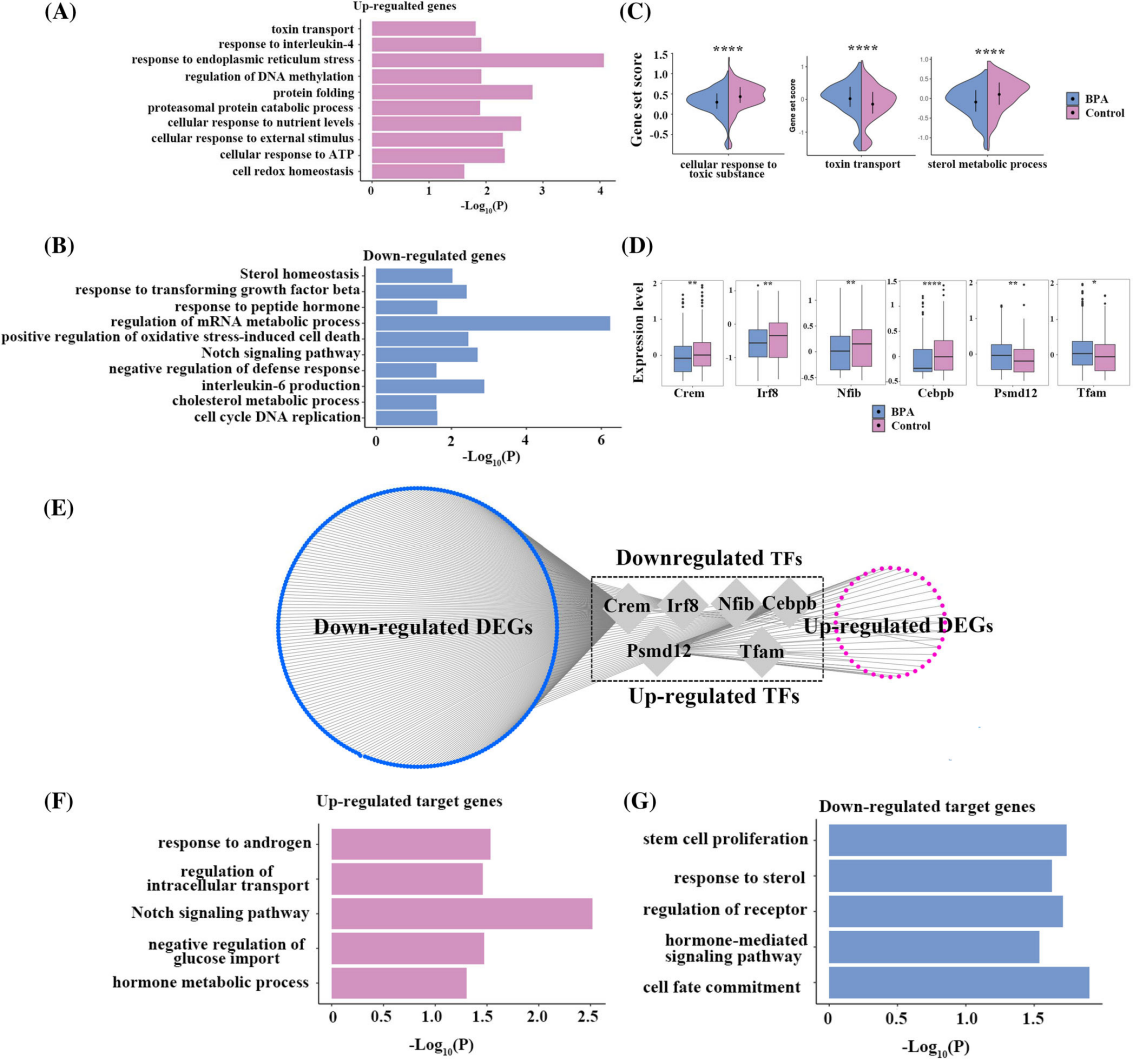
Bisphenol A (BPA) ‐related molecular alterations in Leydig cell. (A–B) Bar plot showing GO terms of up‐regulated (A) and down‐regulated (B) differentially expressed genes in the Leydig cell. (C). Violin plots showing gene set scores of Leydig cell of BPA and Control groups. (D) Box plots showing differentially expressed transcription factor (TF) Crem, Irf8, Nfib, Cebpb, Psmd12 and Tfam between BPA and control groups. (E) Network plot showing the differentially expressed TFs and corresponding differentially expressed target genes in Leydig cell. (F–G) Bar plot showing GO terms of the differentially expressed TFs and corresponding up‐regulated (F) and down‐regulated (G) target genes in Leydig cell. Pink colour showed up‐regulated and blue colour show down‐regulated genes. Data were present as mean ± SEM of three control and three BPA treated testis; *p value  <  .05 and FC > 10% indicate significantly differentially expressed genes and significant GO terms. *p < .05; **p < .01; ***p < .001; ****p < .0001

In conclusion, our results provide the first comprehensive datasets of BPA‐associated genes, pathways, TFs and ligand‐receptor interactions for the major cell types in the testis. Our results highlighted shared or cell type‐specific known and previously unappreciated key molecular processes underlying BPA‐induced perpetual injury in mouse testes. For instance, apart from well‐established ‘steroid metabolism pathway^10^’ in Leydig cells, we identified multiple pathways including down‐regulated pathways in ‘regulation of mRNA metabolic process’ and ‘notch signaling’ may be involved in BPA‐induced testicular toxicity. We expect that beyond the valuable exploration of BPA‐associated signatures and novel insights regarding BPA‐induced perpetual injury, our ScRNA‐seq results will provide a useful resource and a novel angle for studying other related environmental toxicants that affect the male reproductive system. Overall, our findings will advance a variety of efforts toward understanding BPA‐induced testicular injury in mice and providing novel insight into male infertility.

## CONFLICT OF INTEREST

The authors have declared that there is not any conflict of interest.

## Supporting information

Supporting Information

Supporting Information
