# Supplementary material for: A single‐cell atlas of bisphenol A (BPA)‐induced testicular injury in mice
Source: Clin Transl Med. 2022 Mar 28;12(3):e789. doi: 10.1002/ctm2.789 (PMC8958349; doi:10.1002/ctm2.789)
Supplement: Supplementary file 1 — Supporting Information [file CTM2-12-e789-s001.docx]

**Supplementary Data Set**

**MATERIALS AND METHODS**

**Animals and BPA treatment**

This study was carried out in strict accordance with the Guidelines of the institution’s Animal Care and Use Committee. All C57BL/6 mice (male, postnatal day 21, ordered from Charles River in China) were housed under a 12 light-dark cycle and provided with food and water ad libitum. For drug treatment, mice were given BPA (dissolved in corn oil) at the concentration of 10 mg/kg bw/d by intraperitoneal injection for three weeks. The dosage used in this study was referred to environmental relevance, human concentrations, and previous findings, and our selected dose is comparable or less than those in previous toxicological studies.^1-5^ Notably, 10 mg/kg/d a day of BPA was used in our study. The dosage is relevant and consistent with previous studies that suggested that below 50 mg/kg/day was regarded as “low dosage” in laboratory exposures with mammalian animal models.^6^ However, future studies using lower concentrations to systematically characterize BPA-induced testicular injuries are warranty.

**Hematoxylin and eosin (H&E) staining**

After fixation, the testicular tissues (N=5) were embedded and then serially sliced into 4 μm sections and subjected to H&E staining. Images were observed and captured with a microscope.

**Preparation of single-cell suspension**

Single-cell suspension was prepared according to a previous report.^7^ Briefly, testes of mice were excised. Seminiferous tubules were then digested using Collagenase IA (Sigma), and DNase I (Thermo), and trypsin at 37 degrees. Cells filtered through a 100 mm strainer, washed with PBS, and re-suspended in MACS buffer containing 0.5% BSA, and single-cell suspensions were used directly for Drop-Seq.

**Single-cell libraries preparation and sequencing**

Drop-seq sequencing libraries were established according to the previously described protocol.^8^ The single cells suspension and beads were diluted and mixed to co-encapsulation occupancy of 0.05. Individual droplets were harvested, and mRNA was reverse transcribed before cDNA was amplified. A 3ʹ gene expression library was then constructed using a 10 x Genomics Chromium Single Cell system using v3 reagent Kit according to the manufacturer’s instructions. Sequencing was performed on a NovaSeq 6000 (Illumina) by Novogene (China).

**Read alignment and gene expression quantification**

Reads were aligned to the mm10 reference genome obtained from Ensembl. Gene annotations were derived from the GENCODEvM20. 10 x Genomics Cell Ranger 6.1.1 was used to perform alignment, unique molecular identifiers (UMI) de-multiplexing, and cell barcode identification, generating a cells x genes count matrix.

**Quality control**

Calculation of the single-cell expression matrix generated by Cell Ranger was performed using the Seurat package (version 3.6.1) for filtering, data normalization, dimensionality reduction, clustering, and gene differential expression analysis. Cells with less than 300 genes detected or a mitochondrial gene ratio of greater than 20% were excluded. Doublets were detected using the DoubletFinder package (version 2.0.2).^9^ The mean-variance-normalized bimodality coefficient (BCMVN) of each sample was calculated to determine the neighborhood size (pK), and the number of artificial doublets (pN) was set to 0.25. Doublets were then detected.

**Clustering and cell-type identification**

For each cell, the counts were logged normalized (1 + counts per 10,000). Variable genes were selected with the default parameters. Variable genes were projected into a low-dimensional subspace using canonical correlation analysis (CCA) across samples to correct batch effects. A shared nearest neighbor graph was constructed based on the Euclidean distance in the low-dimensional subspace spanned by the selected significant principal components. Cells were clustered at an appropriate resolution. Cells were visualized using a Uniform Manifold Approximation and Projection for Dimension Reduction (UMAP) algorithm. Differential expression analysis for each cluster was performed using the Wilcoxon rank-sum test. Cell types were assigned to each cluster using the abundance of known marker genes. Through the above pipeline, we processed the scRNA-seq data of 77776 high-quality cells from the testis of mice.

**Differential genes expression analyses**

Differential genes (DEGs) expression analysis for each cell type between different groups was performed using the non-parametric two-sided Wilcoxon rank-sum test and only those with |LogFC| > 0.1, p value < 0.05 were considered to be BPA-associated DEGs.

**Gene Ontology enrichment analysis**

Enrichr was used to perform gene set enrichment analysis against the Gene Ontology Biological Process 2018 version gene set collection. MSigDB Hallmark gene sets^10^ were used to compute enrichment scores using Fisher’s exact test. In both cases, we corrected multiple hypothesizes testing using the Benjamini-Hochberg procedure. Results were visualized with the ggplot2 R package (https://ggplot2.tidyverse.org/) (version 3.2.1).

**Transcriptional regulatory network analysis**

Transcriptional regulatory network analysis was conducted using SCENIC workflow (version 1.1.2.2) with default parameters based on mm10 database from RcisTarget (version 1.6.0).^11^ For BPA-related transcriptional regulatory networks, only BPA-related DEGs were used as input for transcriptional regulator inferring. All selected cell types were calculated together. The transcription regulatory network obtained was visualized by the ggraph R package.

**Cell-cell communication analysis**

Cell-cell communication analysis was performed using the CellChat (version 1.1.0) as described previously.^12^ Only receptors and ligands expressed in more than 10% cells of any cell types from either untreated or BPA-treated samples were further evaluated. We randomly permute the cluster labels of all cells 1,000 times and determine the mean of the average receptor expression level in a cluster and the average ligand expression level in the interacting cluster. For each receptor–ligand pair in each pairwise comparison between two cell types, this generates a null distribution. By calculating the proportion of the means which are as or higher than the actual mean, we obtain a P value for the likelihood of cell-type specificity of a given receptor–ligand complex. Only those with a p value < 0.01 were used for the prediction of cell-cell communication between any two cell types.

**Statistical analyses**

All data were statistically analyzed using a two-tailed t-test to compare differences between different groups, assuming equal variance with R package. P values were presented for bioinformatics analyses. *P* value < 0.05 was considered statistically significant.

**Availability of data and materials**

The data reported in this paper have been deposited in the OMIX, China National Center for Bioinformation / Beijing Institute of Genomics, Chinese Academy of Sciences (https://ngdc.cncb.ac.cn/omix: accession no.OMIX791).

**References**

1. Cao T, Cao Y, Wang H, Wang P, Wang X, Niu H*, et al*. The Effect of Exposure to Bisphenol A on Spermatozoon and the Expression of Tight Junction Protein Occludin in Male Mice. *Dose Response* 2020; **18**: 1559325820926745.

2. Ahbab MA, Barlas N, Karabulut G. The toxicological effects of bisphenol A and octylphenol on the reproductive system of prepubertal male rats. *Toxicol Ind Health* 2017; **33**: 133-46.

3. Bansal A, Rashid C, Xin F, Li C, Polyak E, Duemler A*, et al*. Sex- and Dose-Specific Effects of Maternal Bisphenol A Exposure on Pancreatic Islets of First- and Second-Generation Adult Mice Offspring. *Environ Health Perspect* 2017; **125**: 097022.

4. Michałowicz J. Bisphenol A--sources, toxicity and biotransformation. *Environ Toxicol Pharmacol* 2014; **37**: 738-58.

5. Nah WH, Park MJ, Gye MC. Effects of early prepubertal exposure to bisphenol A on the onset of puberty, ovarian weights, and estrous cycle in female mice. *Clin Exp Reprod Med* 2011; **38**: 75-81.

6. Richter CA, Birnbaum LS, Farabollini F, Newbold RR, Rubin BS, Talsness CE*, et al*. In vivo effects of bisphenol A in laboratory rodent studies. *Reprod Toxicol* 2007; **24**: 199-224.

7. Green CD, Ma Q, Manske GL, Shami AN, Zheng X, Marini S*, et al*. A Comprehensive Roadmap of Murine Spermatogenesis Defined by Single-Cell RNA-Seq. *Dev Cell* 2018; **46**: 651-67.e10.

8. Macosko EZ, Basu A, Satija R, Nemesh J, Shekhar K, Goldman M*, et al*. Highly Parallel Genome-wide Expression Profiling of Individual Cells Using Nanoliter Droplets. *Cell* 2015; **161**: 1202-14.

9. McGinnis CS, Murrow LM, Gartner ZJ. DoubletFinder: Doublet Detection in Single-Cell RNA Sequencing Data Using Artificial Nearest Neighbors. *Cell Syst* 2019; **8**: 329-37.e4.

10. Liberzon A, Birger C, Thorvaldsdóttir H, Ghandi M, Mesirov JP, Tamayo P. The Molecular Signatures Database (MSigDB) hallmark gene set collection. *Cell Syst* 2015; **1**: 417-25.

11. Aibar S, González-Blas CB, Moerman T, Huynh-Thu VA, Imrichova H, Hulselmans G*, et al*. SCENIC: single-cell regulatory network inference and clustering. *Nat Methods* 2017; **14**: 1083-6.

12. Jin S, Guerrero-Juarez CF, Zhang L, Chang I, Ramos R, Kuan CH*, et al*. Inference and analysis of cell-cell communication using CellChat. *Nat Commun* 2021; **12**: 1088.
