# Supplementary material for: A single‐cell atlas of bisphenol A (BPA)‐induced testicular injury in mice
Source: Clin Transl Med. 2022 Mar 28;12(3):e789. doi: 10.1002/ctm2.789 (PMC8958349; doi:10.1002/ctm2.789)
Supplement: Supplementary file 2 — Supporting Information [file CTM2-12-e789-s002.docx]

**
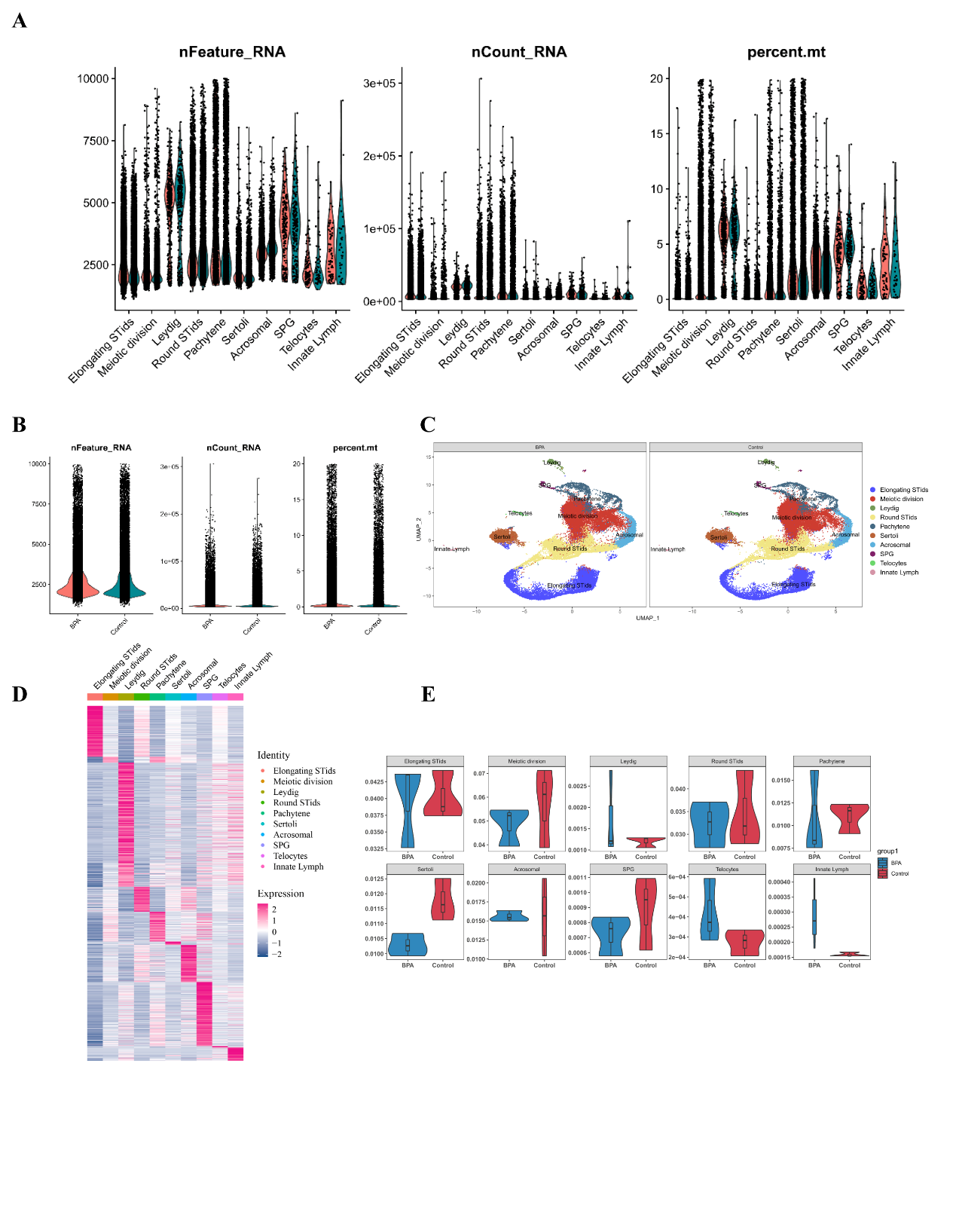
**

**Fig. S1 Single-cell transcriptome analysis of the testis upon BPA treatment**

**A**. Distribution profiles of per-cell attributes compared across different cell types in the testis.

**B**. Distribution profiles of per-cell attributes compared in untreated and BPA-treated samples.

**C**. UMAP plot showing different cell clusters identified based on characteristic markers in testes of untreated and BPA-treated mice.

**D**. Heatmap of cell markers in different cell types.

**E**. Violin plots of cell composition in untreated and BPA-treated mice.

**
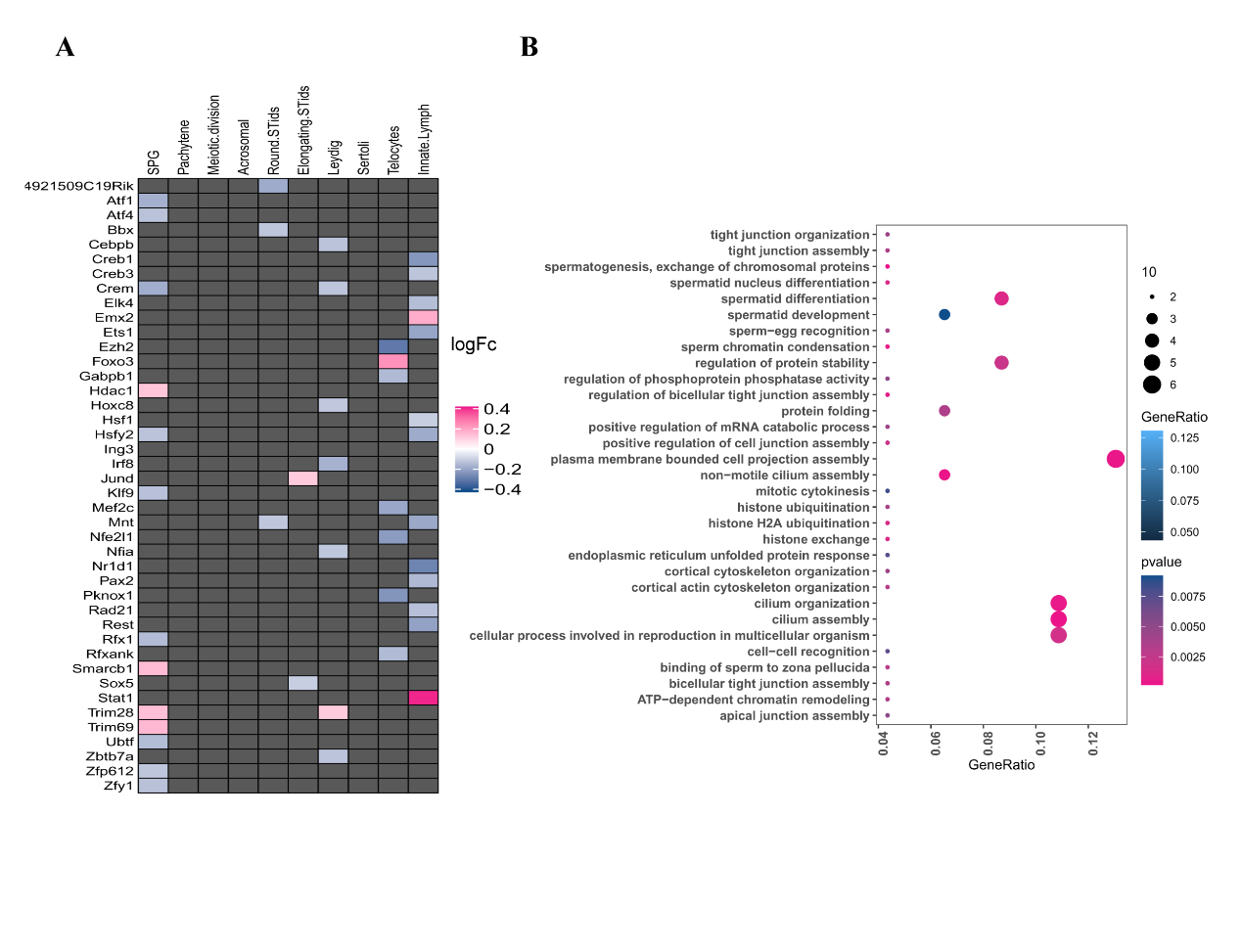
**

**Fig. S2 Predicted changes in transcription factors (TFs) and networks of their targeted genes upon BPA-treatment.**

**A**. Heatmap showing cell-type-specific upregulated or downregulated TFs upon BPA treatment. TFs in red are significantly upregulated and TFs in blue are significantly downregulated. Black genes are not significantly changed upon treatment. (FDR < 0.05 and FC > 10%)

**B.** Dot plot of gene enrichment analysis showing top significantly changed the biological process of BPA-associated TFs in the testis. Color intensity indicates the changed levels (FDR < 0.05 and FC > 10%); circle size indicates the gene count.
